# Validation of an IGF1 Screening Method for Retinopathy of Pre-maturity

**DOI:** 10.3389/fped.2020.615716

**Published:** 2020-12-14

**Authors:** Alejandro Pérez-Muñuzuri, Olalla López-Suárez, Natalia Mandiá-Rodríguez, Carolina López-Sanguos, María José Blanco-Teijeiro, María Luz Couce

**Affiliations:** ^1^Neonatology Unit, University Clinical Hospital of Santiago, Santiago, Spain; ^2^Department of Forensic Sciences, Pathology, Gynecology and Obstetrics, and Paediatrics, Health Research Institute of Santiago (IDIS), University of Santiago de Compostela, Santiago de Compostela, Spain; ^3^Retinal Unit, University Clinical Hospital of Santiago, Santiago, Spain; ^4^Department of Ophthalmology, Health Research Institute of Santiago (IDIS), University of Santiago de Compostela, Santiago de Compostela, Spain

**Keywords:** retinopathy of pre-maturity, pre-term, IGF1, screening, validation

## Abstract

Retinopathy of pre-maturity (ROP) is a retinal disease that causes arrest of vascularization of the retina and can result in retinal detachment and blindness. Current screening protocols may not be sufficiently accurate to identify all at-risk patients. The aim of this study is to validate a method for improved identification of newborns at risk of ROP. We conducted a prospective clinical trial of pre-term newborns <32 weeks of gestation and/or <1,500 g birth weight during a 6-year period in a tertiary care hospital. We applied our new method based on measurement of insulin-like growth factor 1 (IGF1) levels at 3 weeks of age and the presence of sepsis during the first 3 weeks of life. Our screening protocol allowed exclusion of 121 (79.1%) patients for whom American Academy of Pediatrics (AAP) guidelines recommended screening, had a negative predictive value of 100%, and correctly identified all patients with ROP. Following retrospective assessment of our data based on these findings, we propose further restriction of the current AAP indications for screening to <1,100 g and <28 weeks of gestation in order to improve diagnostic efficacy while ensuring optimal use of restriction of human and material resources.

## Introduction

Improvements in management protocols and available therapies have led to increases in both the number and survival of pre-term newborns, and a parallel increase in the prevalence of associated health issues. One such pathology is retinopathy of pre-maturity (ROP), which can negatively affect subsequent development and limit autonomy in later life. ROP, also known as retrolental fibroplasia, is a neovascularization disease, the most severe forms of which can result in retinal detachment and blindness (1, 2). A wide range of factors have been implicated in the etiology of ROP (3–15), including lower gestational age, low birth weight and postnatal weight gain, ethnicity, oxygen treatment, hypercapnia, concomitant diseases (sepsis, bronchopulmonary dysplasia, intraventricular hemorrhage), acidosis, transfusions, environmental luminosity, anemia, transfusions, erythropoietin treatment, and vaginal delivery. Of these, the clearest associations are demonstrated for low gestational age, low birth weight, and oxygen treatment.

Early diagnosis of ROP is necessary in order to institute appropriate therapies, which have enabled a reduction in the incidence of retinal detachment and have substantially improved morbidity in these patients. The American Academy of Pediatrics (AAP) recommends a systematic and protocolized eye examination for pre-term newborns (16), specifically, newborns of ≤ 1.500 g birth weight or gestational age ≤ 30 weeks, and newborns of 1,500–2,000 g birth weight and a gestational age >30 weeks that have unstable clinical evolution or require respiratory support. The moment at which these examinations should begin varies according to the gestational age of each patient (the lower the gestational age the later the first examination). Generally, the first examination is performed between 4 and 6 weeks of extrauterine life.

Current AAP recommendations for ROP screening and monitoring may exclude patients in developing countries who require screening, while also unnecessarily indicating retinal examinations for healthy pre-term babies (particularly in developed countries), resulting in suboptimal use of material and human resources (17, 18). There is therefore a need for improved identification of groups at risk of developing ROP. To better perform individualized screening according to each patient's risk of ROP, it is necessary to understand the pathophysiology of retinopathy. The retina is one of the last organs in the fetus in which vascularization occurs. Vascularization starts at 16 weeks of gestation via angiogenesis, which begins at the papilla of the optic nerve and extends to the periphery of the retina. The angiogenic phenomenon occurs in response to physiological hypoxia, which is triggered by an increase in the metabolic requirements of the developing retina. This physiological hypoxia induces the release of vascular endothelial growth factor (VEGF), which in combination with the insulin-like growth factor 1 (IGF1) enables appropriate vascularization of the retina (19–22). Retinal vascularization is completed by 36–40 weeks of gestation.

The pathophysiology of ROP can be divided into two stages: an initial phase of vascularization arrest, and a second phase of abnormal vasoproliferation. This last phase appears to be triggered by local release of VEGF, an angiogenic factor that accumulates in high concentrations in the retina following hypoxia-induced expression of the VEGF gene (23, 24). VEGF accumulation alone is not sufficient to induce significant vasoproliferation, which also requires the participation of IGF1. IGF1 levels increase progressively with somatic growth and nutritional contribution, reaching a threshold that determines progression to the next phase of ROP pathophysiology (25, 26).

The development and testing of screening systems that are better adjusted to current realities are more than justified given recent advances in our understanding of ROP, the demand for individualized criteria to avoid missed diagnoses through the use of overly general guidelines, and the need to optimize available human and material resources. The main objective of this study was to validate our screening test for ROP in patients >30 weeks of gestational age and >1,250 g birth weight, born between 2013 and 2018, based on our knowledge of the disease and the role of IGF1, and to establish groups with high and low risk of developing ROP. As a secondary objective, we assessed the possibility of applying our screening protocol to patients >28 weeks of gestational age and >1,100 g birth weight, with a view to further restricting the population to which mandatory screening is applied and thereby optimizing available health resources. To this end, we retrospectively studied pre-term babies >28 weeks of gestational age and >1,100 g birth weight from our population to assess the reliability of our method.

Finally, with data collected since 2005, we characterized the progression of IGF1 concentrations between weeks 3 and 5 of life in pre-term newborns with and without ROP in order to better understand the evolution and development of the disease.

## Materials and Methods

### Patients and Study Design

This is a new clinical trial in which we have applied prospectively our screening method to pre-term babies of <1,500 g birth weight and/or <32 weeks of gestational age born at our center between January 2013 and December 2018 with the aim to validate it. Data used to design the screening test were collected prospectively between 2005 and 2012 applying the same inclusion criteria, and have been previously published (27, 28). For patients born before 2005 clinical history data were analyzed retrospectively to identify those with ROP and to identify pre-maturity rates. The study was approved by the Research Ethics Committee of Santiago-Lugo (2012/313) and written informed consent was obtained from the parents or legal guardians of all participants. All methods were carried out in accordance with relevant guidelines and regulations (Declaration of Helsinki).

### Methods

**Figure 1:**
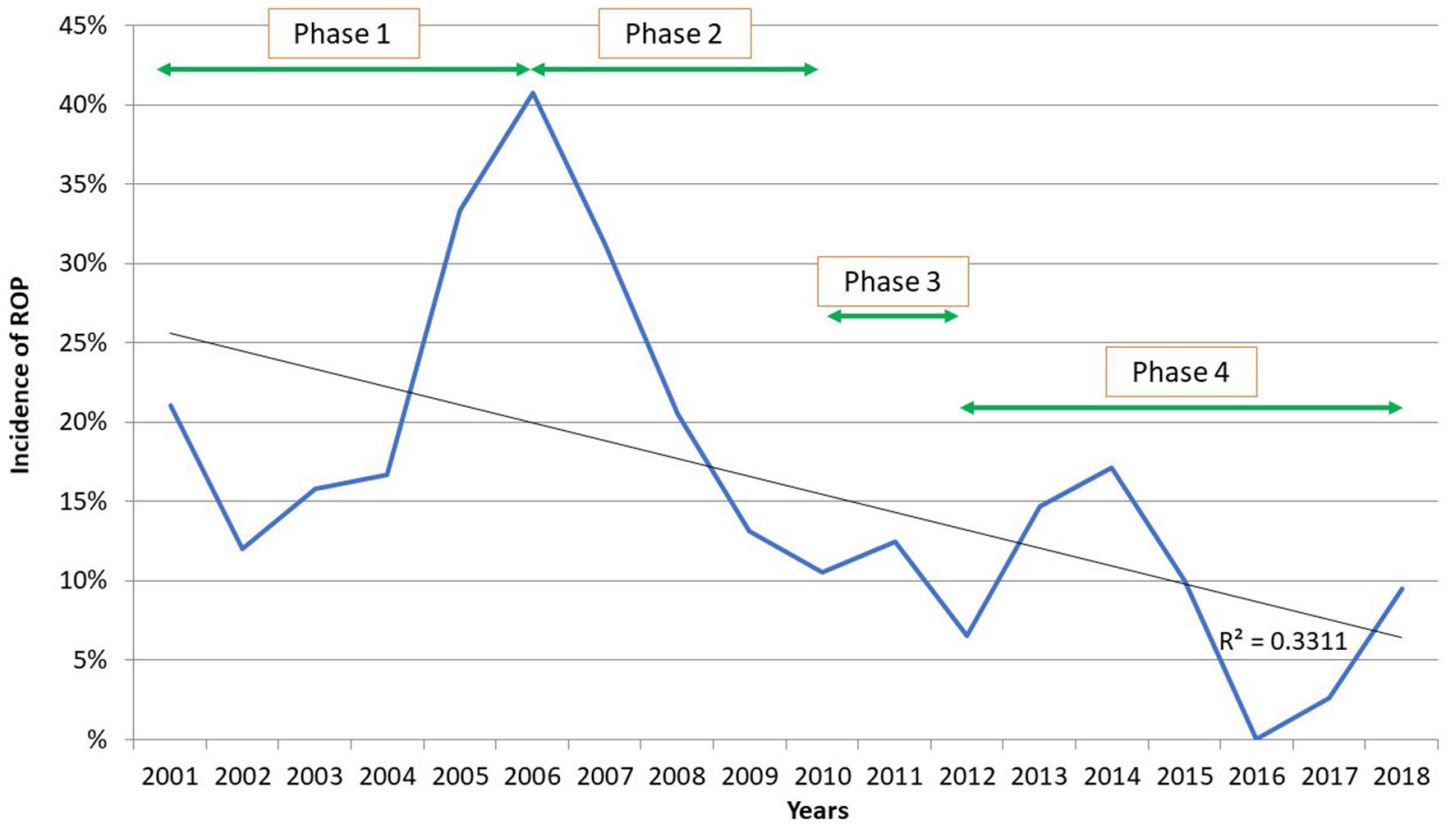
Incidence of ROP in pre-term newborns weighing <1,500 g born in our hospital (2001–2018).

The overall study period can be subdivided into four phases (Figure 1). First, in 2005, in view of the high incidence of ROP and the need for treatment in many newborns and the promising prognostic findings of the ETROP Trial (29), we began to investigate the role of IGF1 in ROP, as well as the etiological factors implicated in the disease.

In the second phase [2005–2010 (27)], we established that patients who develop ROP have lower IGF1 levels over time: this difference is greatest and statistically significant at 3 weeks of age, regardless of gestational age at birth. IGF1 levels <30 ng/ml in the third week of life thus have a discriminative efficacy (as determined by ROC curve analysis) of 87.7% for the diagnosis of ROP. Based on these findings and since this moment (year 2010), we began to optimize the treatments used in all our pre-term babies (strict control of oxygen, mechanical ventilation times, nutrition, and so on) and to identify patients at risk of developing ROP.

In the third phase of the study [2010–2012 (28)], we performed a multiple regression analysis to examine the etiological factors linked to ROP, and demonstrated a significant relationship between IGF1 levels at 3 weeks of age and the presence of neonatal sepsis [defined according to the criteria of the Castrillo Group (30)] during the first 3 weeks of life. Using these variables, retinopathy probability curves were generated to identify high- and low-risk patients and a screening system more selective and restrictive than that of the AAP (16) was established.

The final phase of the study (January 2013 to December 2018) is the reason for this paper. In this stage, our screening method, consisting of more restrictive criteria than those of the current AAP screening protocol, was applied to pre-term newborns in our center in order to validate it.

Our screening method indicates mandatory standard ocular examinations for pre-term babies <30 + 0 weeks of gestation and/or <1,250 g birth weight. Patients that exceed these thresholds are characterized as high or low risk, and are only screened if the probability of ROP as determined by ROC analysis exceeds 30%. To confirm correct retinal vascularization in low-risk unscreened pre-term infants, a single examination is performed at 40 weeks of corrected gestational age. This method has been fully described previously (28).

Data collected since 2005 were used to study the progression of IGF1 levels between weeks 3 and 5 of life in order to better understand the development of the disease.

### Statistical Analysis

Data were analyzed using the SPSS® 20.0 for Windows® statistical package. The quantitative variables were analyzed using the Student's *t*-test and *U*-Mann–Whitney according if they were normalized or not. Qualitative variables were compared using the Chi-squared test. An ANOVA test was used to compare more than two quantitative variables. A *p* <0.05 was considered statistically significant.

## Results

### Global Study (Phase 1–4)

A total of 882 pre-term infants of <1,500 g birth weight and/ or <32 weeks of gestation were born at our center between 2001 and 2018. Of these, 608 weighed <1,500 g. The incidence of ROP (proliferative and not) was highest (Phase 1) in those born in 2005 and 2006 (33.33 and 40.74%, respectively), and subsequently decreased progressively in those born later (Figure 1).

### Present Study (Phase 4): Validation Screening Test for ROP in Patients >30 Weeks of Gestational Age and >1,250 g Birth Weight

During the study period (January 2013–December 2018) a total of 153 pre-term infants aged 30–32 weeks of gestation and weighing 1,250–1,500 g were screened at 3 weeks of age to determine their probability of developing ROP. Of these, 32 (20.9%) were deemed high-risk, and underwent ocular examinations, carried out in accordance with AAP recommendations (16). Three patients (9.4%) were diagnosed with ROP; all cases were non-proliferative and resolved spontaneously without treatment. Of the 153 pre-term infants 121 (79.1%) were deemed low-risk; in these patients no ROP was detected and complete retinal vascularization occurred by 40 weeks of gestational age. Our screening method showed 100% sensitivity (Ss), 80.7% specificity (Sp), a positive predictive value (PPV) of 9.4%, and a negative predictive value (NPV) of 100%.

A theoretical retrospective assessment was also conducted during the same period to determine the probability of developing ROP in all patients aged 28–32 weeks of gestation and weighing 1,100–1,500 g (*n* = 202). Of these 202 newborns 51 (25.2%) were high-risk patients, of which 6 (11.8%) had a diagnosis of ROP (all non-proliferative); and 151 (74.8%) were low-risk patients, none of which had ROP (Ss, 100%; Sp, 77.1%; PPV, 11.8%; NPV, 100%). Demographics and characteristics of enrolled infants are showed in Table 1.

**Table 1:** Verification of our screening method for ROP: **(A)** Prospective analysis **(B)** Theoretical retrospective analysis (after expansion of gestational age and weight criteria).

		**(A) 30–32 wk and 1,250–1,500 g (*****n*** **=** **153)**			**(B) 28–32 wk and 1,100–1,500 g (*****n*** **=** **202)**		
		**Low risk (*n* = 121)**	**High risk (*n* = 32)**	***P***	**Low risk (*n* = 151)**	**High risk (*n* = 51)**	***P***
GA (weeks)		31.8	31.41	NS	31.35	30.63	0.015
Birthweight (g)		1,606	1,485	0.001	1,532	1,390	0.000
Length at birth (cm)		41.53	40.44	0.003	40.93	39.8	0.001
Cranial circumference at birth (cm)		29.52	28.95	0.017	29.13	28.05	0.037
Sex	Female	67 (55.4%)	18 (56.2%)	NS	84 (55.6%)	31 (60.8%)	NS
	Male	54 (44.6%)	14 (43.8%)		67 (44.4%)	20 (39.2%	
Type of birth	Vaginal	32 (26.4%)	10 (31.2%)	NS	41 (27.2%)	13 (25.5%)	NS
	Cesarean	89 (73.6%)	22 (68.8%)		110 (72.8%)	38 (74.5%)	
Surfactant treatment		18 (14.9%)	10 (31.2%)	0.033	28 (18.5%)	14 (27.5%)	NS
Respiratory support		105 (86.8%)	24 (75%)	NS	133 (88.7%)	42 (82.4%)	NS
Oxygen administration		32 (27.4%)	11 (34.4%)	NS	47 (32.6%)	16 (31.4%)	NS
Intracranial hemorrhage		1 (0.8%)	1 (3.1%)	NS	4 (2.7%)	1 (2%)	NS
Patent ductus arteriosus		17 (14%)	5 (15.6%)	NS	28 (18.7%)	9 (17.6%)	NS
Bronchopulmonary dysplasia		4 (3.3%)	3 (9.4%)	NS	12 (7.9%)	7 (13.7%)	NS
EPO treatment		0 (0%)	2 (6.2%)	0.006	0 (0%)	7 (13.7%)	0.000
Blood transfusion		2 (1.7%)	2 (6.2%)	NS	3 (2%)	3 (5.9%)	NS
IGF1 3w (ng/ml)		51	30.48	0.000	50.42	27.48	0.000
Sepsis ≤ 3w		8 (6.6%)	5 (15.6%)	NS	10 (6.6%)	9 (17.6%)	0.009
ROP		0 (0%)	3 (9.4%)	0.001	0 (0%)	6 (11.8%)	0.000
		Ss 100%			Ss 100%		
		Sp 80.7%			Sp 77.1%		
		PPV 9,4%			PPV 11.8%		
		NPV 100%			NPV 100%		

*n, number of patients; NS, not significant; Respiratory support includes invasive and not invasive mechanical ventilation and high nasal flow (more than 2 lpm)*.

**Figure 2:**
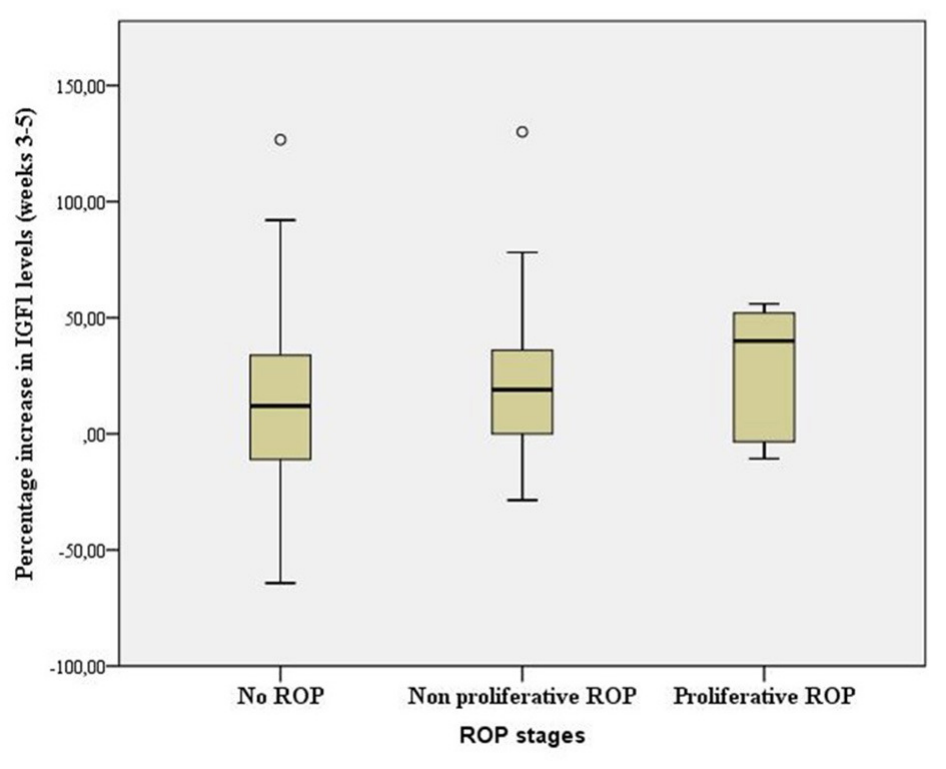
Box plot showing range, quartile 1, quartile 3, and median percentage increase in IGF1 levels between weeks 3 and 5 of life according to ROP grade (*p* > 0.05, ANOVA).

Finally, we studied the progression of IGF1 levels between weeks 3 and 5 of life and their relationship with the grade of ROP in order to better understand the role of IGF1 in the pathophysiology of this disease. We observed an association between greater percentage increases in IGF1 levels -calculated as the increase in IGF1 levels between weeks 3 and 5 of life in relation to the levels existing in the 3rd week- and the risk of developing proliferative ROP [28.97%, CI 95% (−1.18–59.13)]. In patients without ROP [14.10%, CI 95% (6.91–21.29)] or with non-proliferative ROP [21.47%, CI 95% [8.76–34.17)] we observed much lower percentage increases in IGF1 levels. While these associations were not statistically significant a clear trend was evident (Figure 2).

## Discussion

In medicine, control of any disease is based on knowledge of the underlying pathophysiology and the associated etiological variables, which in turn depend on the surrounding environment; the pathogenic power a given disease may vary depending on the place in which it occurs.

A peak in the incidence of ROP in 2005–2006 and the very promising prognostic findings of the Early Treatment for ROP Trial (29), prompted us to begin an exhaustive study of the etiology of this disease, and to investigate the diagnostic relevance of IGF1 levels. Although the number of pre-term infants of <1,500 g birth weight has increased over the years, the mean incidence of ROP (currently 5%) has decreased to a record low, well below that recorded in the SEN1500 database by the Spanish Society of Neonatology (20% in 2017) (31).

Low levels of IGF1 (<30 ng/ml) in the third week of life, regardless of gestational age at birth, imply a high risk of developing ROP (27). Furthermore, sharp increases in IGF1 levels between weeks 3 and 5 of life increase the risk of developing proliferative ROP, probably due to abnormal vascular growth induced by the interaction between rapidly increasing concentrations of IGF1 and local VEGF. A recently reported association between severe forms of ROP and weight gain acceleration may have implications for the choice of therapy used, and in particular for the time window during which IGF1 supplementation is applied (32). Based on these findings it can be hypothesized that low IGF1 levels induce ROP but that rapid increases in IGF1 levels favor more severe forms. Although not statistically significant, our findings demonstrating an association between IGF1 increases and proliferative ROP support this view.

The results of a study based on IGF1 replacement in pre-term infants have been recently published, showing no benefit in the development of ROP (33). Our studies (27, 28) have found a temporal relationship between low levels of IGF1 in the third week of life and the development of ROP, in the same way that a sudden increase between the third and fifth week of life seems to favor the appearance of proliferative phases of the disease. This is why, in our opinion, IGF1 replacement may be useful in the first weeks of life, but beyond the third week of life it can be harmful.

In our hospital, measurement of IGF1 levels in pre-term infants at weeks 3 and 5 of life is performed in parallel with other blood draws as required according to the monitoring protocol for very pre-term infants. It has allowed us to identify patients at high risk of developing ROP and to establish preventive measures based fundamentally on the principles of minimum invasiveness and minimal manipulation. These measures include reducing mechanical ventilation times, optimizing the range of target oxygen saturation (90–95%), improving nutritional contributions (early parenteral nutrition with high protein intake and early initiation of enteral nutrition, promoting breastfeeding or the use of donated human milk), modifying the criteria for central venous channeling, restricting umbilical arterial catheterization to the extent possible, implementing restrictive criteria for the preventive use of erythropoietin, and establishing infection screening systems (zero bacteremia).

Most importantly, the study of retinopathy in our center has led to widespread awareness of the problem by all health personnel (medical doctors and nurses) and has undoubtedly contributed to better control and a reduced incidence of this disease.

Current AAP screening criteria are of low diagnostic utility: large numbers of patients undergo ophthalmological examinations but less than half will be diagnosed with ROP and <10% will have a severe form (type 1 ROP) that will require treatment (34). Our screening protocol is designed to rule out healthy patients who will not develop forms of ROP, ensuring screening of all at-risk patients and very pre-term infants (i.e., those most likely to develop type 1 ROP).

Our screening system allows a reduction in the total number of patients that undergo eye examinations. This in turn allows us to optimize human and material resources and to make economic savings without reducing the quality of care, all while reducing the incidence of retinopathy. Moreover, the screening system has a negative predictive value of 100%, meaning that we can be 100% certain that pre-term patients deemed low risk will not develop the disease. The results of our theoretical retrospective assessment indicate that further restriction of the population that undergoes screening is possible. Based on these findings we could restrict mandatory screening to pre-term newborns <28 + 0 weeks and/or <1,100 g bodyweight: above these cut-off points, screening will be only applied to high-risk patients with a probability of developing ROP >30% as determined by our probability curves.

Several other methods have been developed to screen newborns and identify populations at risk of developing ROP (35–37). Perhaps the best known is WINROP (35). Developed by the Sahlgrenska Center for Pediatric Ophthalmology Research in Sweden, this method is based on the weight progression of pre-term infants. However, there is a poor correlation between IGF1 levels and weight progression (28): patients with good weight progression and low IGF1 levels can develop ROP, while others with poor weight progression and high IGF1 levels may not develop the illness. Weight is a poor indicator of the lean mass in pre-term newborns and is significantly influenced by water gain. Moreover, a recent study concluded that pre-term infants with a high birth weight for their gestational age may not be considered at high risk of ROP by WINROP (38).

The G-ROP method for ROP screening was recently published (39) and subsequently validated (34). This method is based on the examination of pre-term babies <28 weeks of gestational age, <1,051 g birth weight, with poor weight gain during the second, third and fourth 10 days of life. This screening protocol must remain active up to 40 days of life to ensure detection of all at-risk patients. Conversely, in our screening protocol a single evaluation during the third week of life allows differentiation of high-risk from low-risk patients, thereby identifying patients who require a retinal exam. Low weight gain during the second, third and fourth 10 days of life correlates with low IGF1 levels, which we consider an indicator of ROP risk.

The main limitation of the study is that although our initial studies included patients with proliferative and non-proliferative ROP (27, 28), allowing us to determine an IGF1 cut-off point for the risk of developing the disease, in the current validation of the screening method in children over 28 weeks, no proliferative ROP has been detected, so we cannot assure its validity in these patients, although we understand that if it is capable of detecting the mild and incipient forms of the disease, it should also be able to detect serious forms.

Preterm babies in our study were mainly white, and only 3 patients were black. Although studies have linked maternal ethnicity with risk of ROP (40), Reddy et al. proposed that any IGF1-based screening method should take into account maternal ethnicity, given that black patients have been shown to have lower IGF1 levels independent of the risk of developing ROP (41). However, those authors studied only 36 patients and measured IGF1 between 31 and 33 weeks of postmenstrual age. We previously reported that IGF1 sensitivity and specificity peak during the third week of life, independently of gestational age, with a tendency to equalize IGF1 levels throughout the weeks of life, regardless of whether ROP develops or not (27).

Our method is highly effective in our specific clinical environment, significantly reducing the incidence of ROP, but further studies will be necessary to determine its applicability to other hospitals, both in Spain and elsewhere.

In conclusion, our new screening method, based on measurement of IGF1 levels at 3 weeks of life and the presence of sepsis during the first 3 weeks or life, allows restriction of the current AAP screening criteria, probably limiting screening to pre-term babies of <28 weeks of gestation and/or <1,100 g birth weight.

## Data Availability Statement

The raw data supporting the conclusions of this article will be made available by the authors, without undue reservation.

## Ethics Statement

The studies involving human participants were reviewed and approved by Research Ethics Committee of Santiago-Lugo. Written informed consent to participate in this study was provided by the participants' legal guardian/next of kin.

## Author Contributions

AP-M and MC designed the study, reviewed the publications included in the systematic review, contributed to the acquisition and analysis of the data, and drafted the manuscript. OL-S, NM-R, and CL-S contributed to the acquisition of the data and participated in the critical review of the manuscript. MB-T made the retinal examinations and participated in the critical review of the manuscript. All the authors read and approved the final manuscript.

## Conflict of Interest

The authors declare that the research was conducted in the absence of any commercial or financial relationships that could be construed as a potential conflict of interest.
